# GWAS and bulked segregant analysis reveal the Loci controlling growth habit-related traits in cultivated Peanut (*Arachis hypogaea* L.)

**DOI:** 10.1186/s12864-022-08640-3

**Published:** 2022-05-27

**Authors:** Li Li, Shunli Cui, Phat Dang, Xinlei Yang, Xuejun Wei, Kai Chen, Lifeng Liu, Charles Y. Chen

**Affiliations:** 1 State Key Laboratory for Crop Improvement and Regulation in North China, College of Agronomy, Hebei Agricultural University, Baoding, 071001 The People’s Republic of China; 2 Department of Crop, Soil and Environmental Sciences, Auburn University, Auburn, AL 36948 USA; 3 School of Landscape and Ecological Engineering, Hebei University of Engineering, Handan, 056038 The People’s Republic of China; 4 USDA-ARS National Peanut Research Laboratory, Dawson, GA 39842 USA

**Keywords:** Peanut (*Arachis hypogaea* L.), BSA-seq, GWAS, Plant growth habit

## Abstract

**Background:**

Peanut (*Arachis hypogaea* L.) is a grain legume crop that originated from South America and is now grown around the world. Peanut growth habit affects the variety’s adaptability, planting patterns, mechanized harvesting, disease resistance, and yield. The objective of this study was to map the quantitative trait locus (QTL) associated with peanut growth habit-related traits by combining the genome-wide association analysis (GWAS) and bulked segregant analysis sequencing (BSA-seq) methods.

**Results:**

GWAS was performed with 17,223 single nucleotide polymorphisms (SNPs) in 103 accessions of the U.S. mini core collection genotyped using an Affymetrix version 2.0 SNP array. With a total of 12,342 high-quality polymorphic SNPs, the 90 suggestive and significant SNPs associated with lateral branch angle (LBA), main stem height (MSH), lateral branch height (LBL), extent radius (ER), and the index of plant type (IOPT) were identified. These SNPs were distributed among 15 chromosomes. A total of 597 associated candidate genes may have important roles in biological processes, hormone signaling, growth, and development. BSA-seq coupled with specific length amplified fragment sequencing (SLAF-seq) method was used to find the association with LBA, an important trait of the peanut growth habit. A 4.08 Mb genomic region on B05 was associated with LBA. Based on the linkage disequilibrium (LD) decay distance, we narrowed down and confirmed the region within the 160 kb region (144,193,467–144,513,467) on B05. Four candidate genes in this region were involved in plant growth. The expression levels of *Araip.E64SW* detected by qRT-PCR showed significant difference between ‘Jihua 5’ and ‘M130’.

**Conclusions:**

In this study, the SNP (AX-147,251,085 and AX-144,353,467) associated with LBA by GWAS was overlapped with the results in BSA-seq through combined analysis of GWAS and BSA-seq. Based on LD decay distance, the genome range related to LBA on B05 was shortened to 144,193,467–144,513,467. Three candidate genes related to F-box family proteins (*Araip.E64SW*, *Araip.YG1LK*, and *Araip.JJ6RA*) and one candidate gene related to PPP family proteins (*Araip.YU281*) may be involved in plant growth and development in this genome region. The expression analysis revealed that *Araip.E64SW* was involved in peanut growth habits. These candidate genes will provide molecular targets in marker-assisted selection for peanut growth habits.

**Supplementary Information:**

The online version contains supplementary material available at 10.1186/s12864-022-08640-3.

## Background

Peanut (*Arachis hypogaea* L.) is a grain legume crop that originated from South America and is grown around the world [1]. Peanut seed is rich in oil and is a great source of protein, vitamins, and minerals, and it is added to many foods as a functional ingredient [2]. Peanut has been grown for more than 3,500 years in tropical, subtropical, and warm temperate regions throughout the world [3]. Because of the multiple agroclimatic zones, the characteristics of growth habits, seed, and pod are significant differences. Based on morphology and growth habits, the species *Arachis hypogaea* L. is classified into two subspecies, *A. hypogaea* ssp*. hypogaea* and *A. hypogaea* ssp. *fastigiata*. The subspecies *hypogaea* is further divided into the botanical varieties known as var. *hypogaea* and var. *hirsuta*, while *fastigiata* is further classified into four botanical varieties named var. *fastigiata*, var. *vulgaris*, var. *aequatoriana,* and var. *peruviana* [4]. Among these classifications, the plant type of subspecies *hypogaea* is either prostrate (runner) or erect (bunch), and the plant type of subspecies *fastigiata* is entirely erect [5]. Growth habit, also called plant architecture or plant type, is an important morphological trait affecting crop yield and tillage method. The prostrate or big branch angle plant type presents loose canopy architectures, which are suitable for sparse planting. In contrast, a plant type with an erect or small branch angle can exhibit compact canopy architectures, which are suitable for high-density planting. Accordingly, plant architecture has been a major breeding target for crop improvement. Determination of genetic mechanisms controlling plant type will facilitate architecture improvement in peanut.

Some domestication-related genes of plant architecture, especially the branch angle between the lateral branch and the main stem, have been cloned in crops. In rice, Li et al. showed that the *LAZY1* gene controls the angle of rice tillers, so the loss of function in *LAZY1* will cause the tiller angle to increase [6]. Jin et al. discovered that the *PROG1* gene controls the tiller angle and number of tillers, which makes it an important domestication-related gene that can be used to change rice architecture from prostrate to erect [7]. Wu et al. reported that a 110-kb deletion linked to the *PROG1* gene on the short arm of chromosome 7 promotes the vital transition from the prostrate growth habit of wild rice to the erect growth habit of Asian cultivated rice [8]. Yu et al. (2007) demonstrated that the difference between the *TAC1* and *tac1* gene sequences presents a prostrate and an erect plant architecture, which has a mutation (AGGA → GGGA) in the 3’-splicing site of the fourth 1.5-kb intron in the 3’-untranslated region [9]. Subsequently, the branch angle genes or QTLs have also been identified not only in monocot crops like maize [10], but also in dicots like rapeseed [11], sesame [12], peach [13], pea [14], and tomato [15]. Previous studies of peanut growth habit have revealed a disagreement on whether the inheritance of these traits is cytoplasmic or nuclear [16–20]. Additionally, whether the inheritance mechanism controlling branch angle is monogenic or polygenic remains unclear, as well [21–23]. Although molecular markers have been developed over the past few decades to study the genetic mechanisms of disease-resistance, stress-tolerance, and high yield, there are only several peanut studies dealing with growth habits. Fonceka et al. determined that peanut growth habit is controlled by several QTLs with differing amounts of phenotypic variation, utilizing a chromosomal segment substitution line population[24]. Kayam et al. combined bulk segregant analysis with sequencing results and identified a major QTL for peanut growth habit on B05 within a ~ 1.1 Mb segment [23]. Traditionally, linkage mapping has been an effective method for mapping the regions of a genome with phenotypes in different populations, such as recombinant inbred lines (RIL) and double haploid lines [25, 26]. Moreover, bulk segregant analysis (BSA) offers a method for rapidly identifying genes or genomic regions tightly associated with a given phenotype. For example, two bulks were constructed with a distinct phenotype derailing the allele distribution in each bulk around the target genetic region controlling the traits and genetic differences were identified [27]. With the development of high-throughput genotyping technologies and access to more computational power, combining whole genome sequencing with BSA can be an efficient way to identify QTLs [28]. In addition, GWAS is a quantitative approach based on LD that can associate genotype to specific phenotype in diverse populations [29]. To reduce the false positives generated from different QTL mapping methods, two or more methods can be coupled to capture genotypic information and increase the power to verify associations [30]. Duo et al. identified a candidate gene (*CIFS1*) controlling fruit shape in watermelon, which combined the GWAS profiles among 315 accessions and BSA-seq mapping in the F_2_ population [31]. Mu et al., by using genome-wide linkage mapping and BSA-seq, mapped a wheat stripe rust resistance QTL in a 0.4 cM genetic interval on chromosome 7B [32]. Zhao et al. found a major QTL on LG-F (chromosome 13) for resistance to *Sclerotinia sclerotiorum* via linkage and association mapping in soybean [33]. Sun et al. discovered and validated seven consensus QTLs for seed oil content from GWAS and linkage mapping methods in *Brassica napus* [34]. For peanut, Luo et al. using the BSA-seq method discovered the nine candidate genes in the genomic regions of 2.75 Mb on A09 and 1.1 Mb on B02, which control shelling percentage in peanut [35]. Zhang et al. identified genetic markers associated with the key agronomic trait, such as protein and oil content, by GWAS in peanut based on 268 lines and 120 markers [36]. Zhang et al. analyzed 11 agronomic traits in 158 peanut accessions by GWAS, and 1,429 genes were identified in a 200 k genomic region related to domestication [37]. To date, there are few reports of growth habit-related traits based on QTL-seq in peanut. To identify candidate genes associated with peanut growth habit-related traits, we performed a GWAS analysis using a peanut Affymetrix version 2.0 SNP array and the U.S. mini-core germplasm collection based on phenotypic information in two environments. Two DNA pools with extreme phenotypes in F_2_ population were utilized for BSA-seq. Two methods previously described were deployed to identify the candidate genes associated with growth habit-related traits in peanut. The results may provide a reference for genetic dissection of peanut growth habit-related traits.

## Results

### Phenotypic evaluation of growth habit-related trait

To evaluate the phenotypic variation of growth habit-related traits, five traits for LBA, MSH, LBL, ER, and IOPT in two environments were analyzed. The result showed large phenotypic variation within the U.S. peanut mini-core collection. LBA, MSH, LBL, ER, and IOPT varied from 32.65 to 87.30, 8.50 to 63.40, 15.00 to 77.60, 6.38 to 46.58, and 0.77 to 4.90, respectively (Table 1). The coefficient of variance ranged from 15.38% to 36.89% (Table 1). The ANOVA results based on phenotypic traits in the two environments indicated significant differences among genotypes, environments, and genotype × environment interactions (Additional file 1). Correlations of LBA with LBL, ER, and IOPT were significantly positive, while LBA with MSH was negative in two environments. The correlation between MSH and LBL revealed a significantly positive relationship, while the relationship between MSH, ER, and IOPT revealed negative correlations in two environments. The LBL had a significant positive relationship with ER and IOPT. A positive correlation was also found between ER and IOPT (Additional file 2).

**Table 1:** Phenotypic variation for growth habit-related traits in the U.S. mini-core collection

Environment	Trait	Max	Min	Mean	*SD*	*CV*(%)
Qingyuan	LBA	87.30	36.10	67.85	10.43	15.38%
(China)	MSH	32.50	8.50	18.67	6.42	34.40%
	LBL	49.00	15.00	29.88	7.87	26.33%
	ER	43.63	6.38	21.70	6.59	30.37%
	IOPT	4.90	0.92	1.72	0.63	36.89%
Dawson	LBA	87.30	32.65	68.71	12.80	18.63%
(U.S.)	MSH	63.40	20.38	38.50	9.19	23.87%
	LBL	77.60	26.55	51.97	10.14	19.52%
	ER	46.58	11.75	28.20	7.34	26.03%
	IOPT	2.68	0.77	1.38	0.30	21.52%

*SD, standard deviation; CV is the coefficient of variation*

For the F_2_ population, to investigate the inheritance of LBA in peanut, a total of 548 F_2_ individuals derived from ‘Jihua 5’ × ‘M130’ segregated as 182 prostrate type, 82 erect type, and 286 medium type, which didn’t fit any typical separation ratio of one gene model. Thus, the LBA trait was controlled by multiple genes.

### Genetic variation, population structure and linkage disequilibrium in germplasm population

**Fig. 1:**
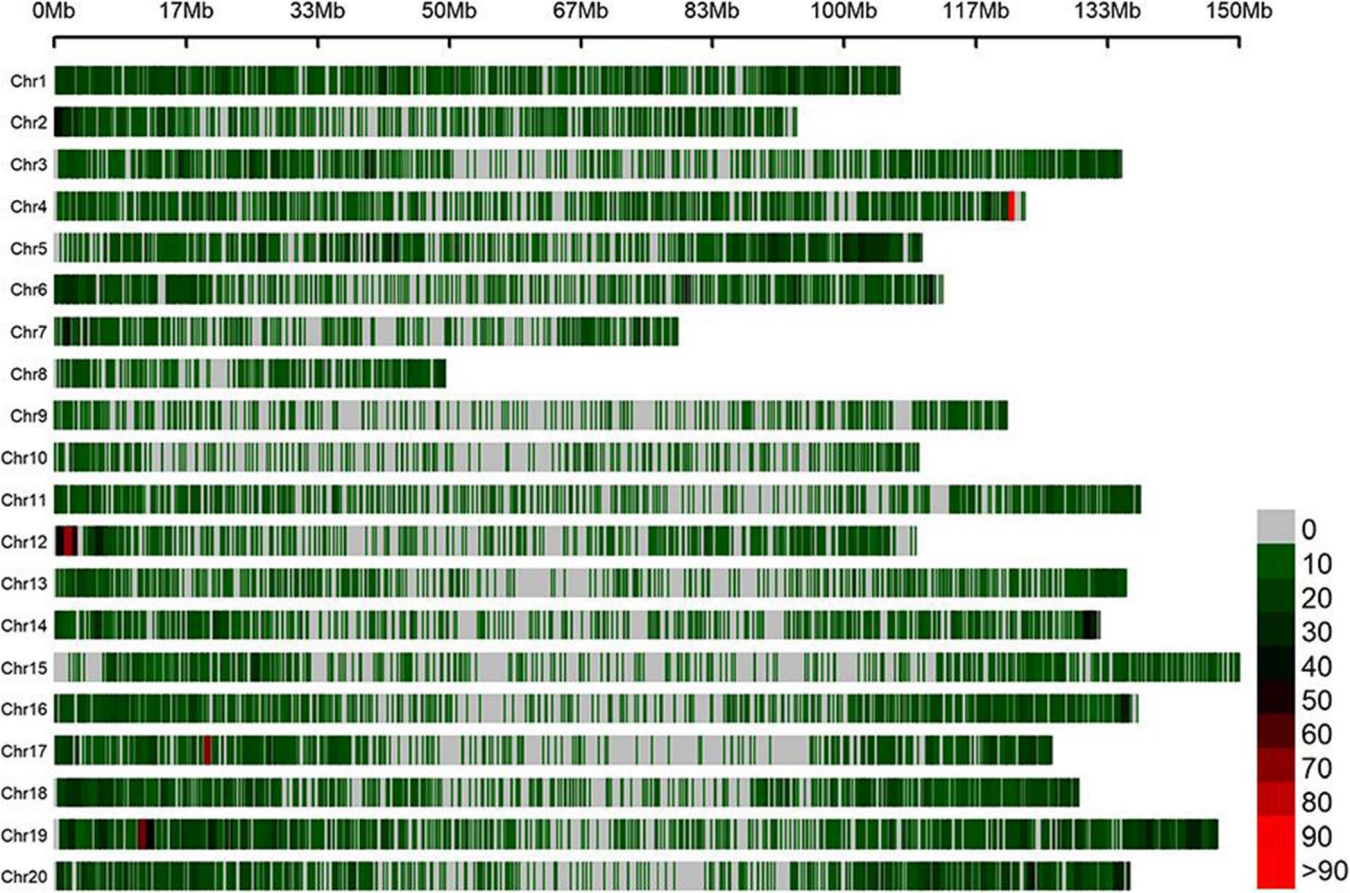
The distribution of SNPs detected in the entire association mapping panel. Red and gray horizontal bars show genomic regions that are rich and poor in SNPs, respectively

The 103 genotypes of the U.S. mini-core collection were examined using the SNP array (Affymetrix) at GeneSeek (Lincoln, Nebraska, USA). A total of 12,342 SNPs markers were screened after filtering out SNPs with low-quality based on a call rate < 0.95 and minor allele frequency < 0.05. The marker density was shown in Fig. 1. Chromosome B09 had the maximum density of SNP (0.10 M/SNP) and the number of SNPs involved with it was 1,428, while chromosome A10 had the minimum density of SNP (0.37 M/SNP) and the number of SNPs involved with it was 293. The polymorphism information content (PIC) values ranged from 0.26 to 0.30 among chromosomes, and the mean PIC was 0.28 (Table 2).

**Table 2:** The summary of the number of polymorphic SNPs mapped in the 20 chromosomes of peanut

Chr	No. of SNPs	Chr. length (Mb)	Density of SNP (kb/SNP)	PIC
A01	845.00	106.85	126.45	0.28
A02	452.00	93.54	206.94	0.29
A03	607.00	134.89	222.23	0.28
A04	637.00	122.71	192.63	0.27
A05	586.00	109.45	186.77	0.27
A06	554.00	112.00	202.16	0.28
A07	440.00	78.82	179.13	0.28
A08	299.00	49.37	165.13	0.28
A09	350.00	120.50	344.28	0.29
A10	293.00	109.30	373.05	0.30
B01	601.00	137.29	228.43	0.28
B02	325.00	108.95	335.22	0.28
B03	482.00	135.54	281.19	0.28
B04	487.00	132.17	271.39	0.29
B05	456.00	149.84	328.61	0.27
B06	633.00	136.16	215.10	0.29
B07	680.00	126.13	185.48	0.30
B08	1010.00	129.56	128.28	0.26
B09	1428.00	147.06	102.99	0.26
B10	1177.00	135.98	115.53	0.26

*Chr* chromosome, *PIC* polymorphism information content

**Fig. 2:**
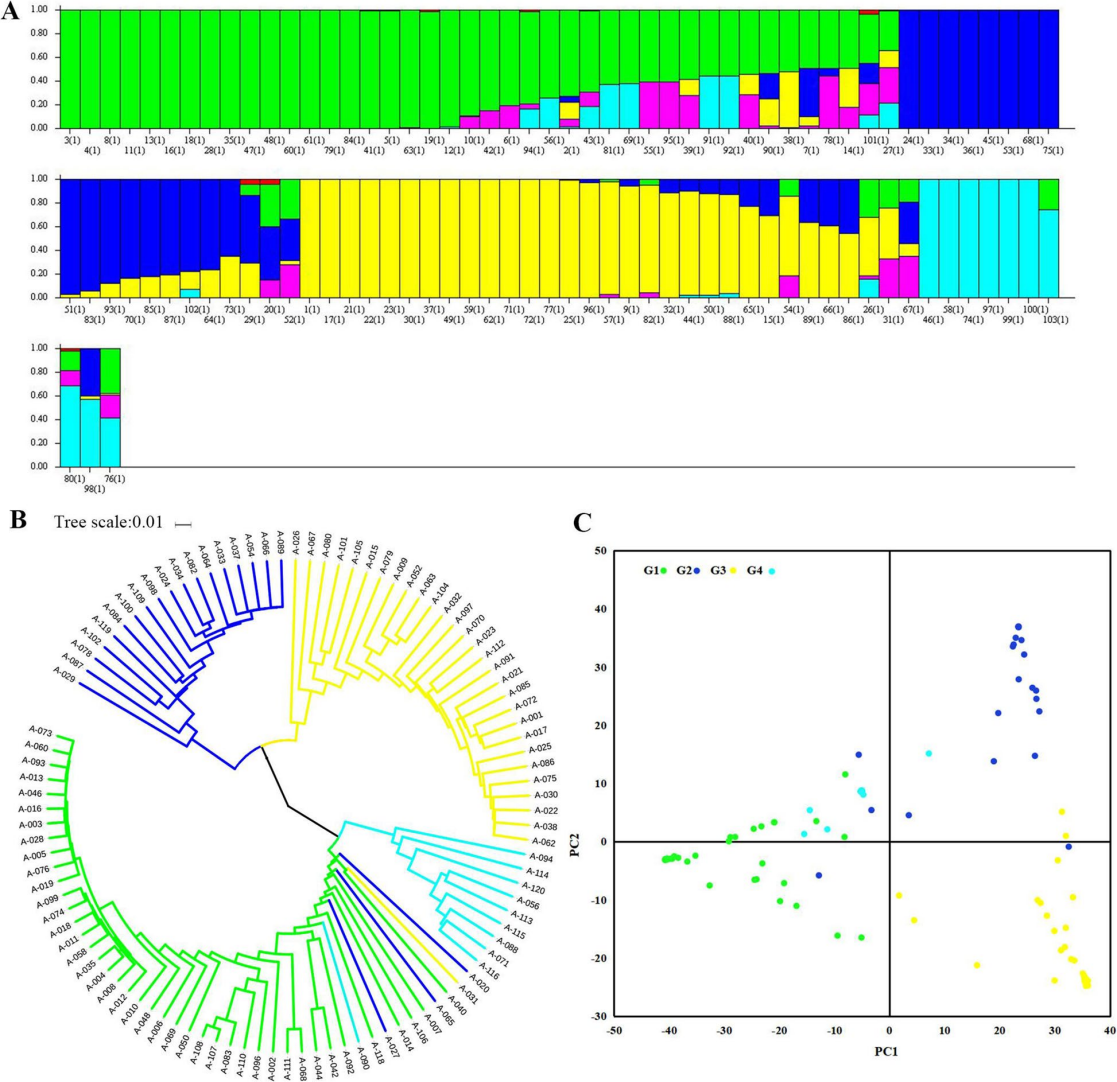
Population structure analysis, phylogenetic tree construction, and principal component analysis (PCA) within the U.S. mini-core collection. **A** Population structure analysis. **B** Phylogenetic tree constructed with UPGMA clustering method. **C** Principal component analysis showing the population structure in the diversity panel. Four subpopulations are designated as G1, G2, G3, and G4

To evaluate the population variation, the analysis of population structure, phylogenetic relationship, and PCA were carried out using the 12,342 filtered SNPs. Structure analysis revealed that the U.S. peanut mini-core collection was clustered into four sub-populations (G1, G2, G3, and G4) (Fig. 2). G1, G2, and G3 demonstrated notable genetic differences, but G4 had no clear genetic differences from G1 and G2 (Fig. 2C). According to the result of the UPGMA tree analysis, the U.S. mini-core collection was also classified into four major clusters.

Among 103 accessions, there were four botanical varieties that were classified based on morphological data collected from the field and current GRIN taxonomy [4]: var. *fastigiata*, var. *hypogaea*, var. *peruviana*, and var. *vulgaris* (Additional file 3)*.* As shown in Additional file 4, the frequency of each botanical variety within each sub-population was presented; 61.82% of *hypogaea* accessions were assigned to G1, 47.06% of *fastigiata* accessions were classified into G2, 64% of *vulgaris* were classified into G3, and 100% of *peruviana* accessions were classified into G4. Despite some discrepancies, the population structure is corresponding to the classification of botanical variety.

LD was estimated from the r^2^ (r^2^ < 0.2 was considered unlinked) correlation between each marker in the 103 accessions of the U.S. mini-core collection. The LD decay in this population was approximately 0.16 M with r^2^ at 0.2 (Additional file 5).

### Generation and analysis of BSA-seq data

**Fig. 3:**
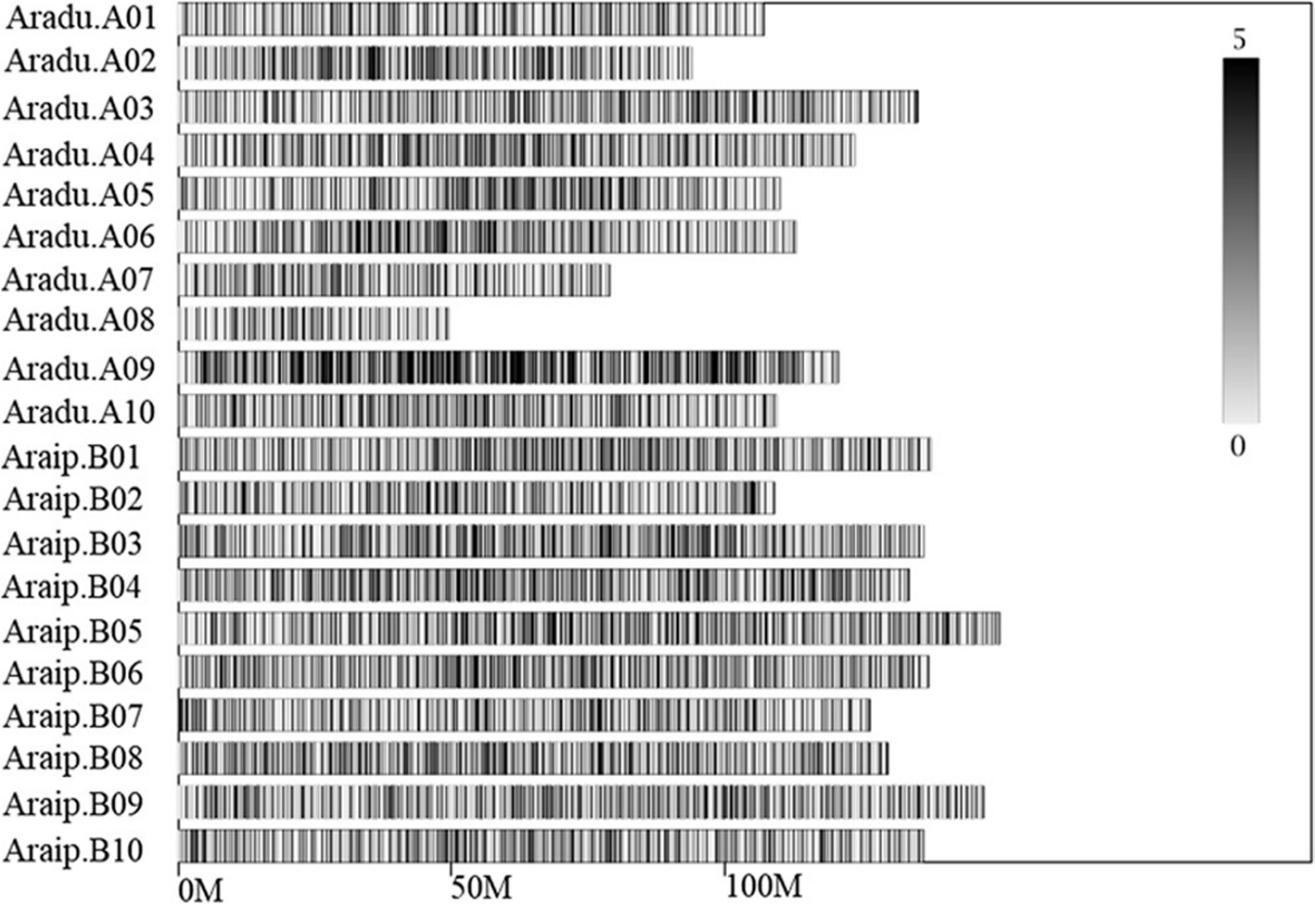
A distribution diagram of the markers on each chromosome. Black and gray horizontal bars show genomic regions that are rich and poor in SNPs, respectively

For the paternal inbred line (‘M130’), 150,190 SLAFs were generated from 3,355,918 reads with an average coverage of 22.34-fold for each SLAF. For the maternal line (‘Jihua 5’), 150,080 SLAFs were produced from 2,673,407 reads, and the average coverage of each SLAF was 17.81-fold. For the analysis of the P-pool, 153,081 SLAFs were screened from 6,595,001 reads in each genotype with an average coverage of 43.08-fold. For the analysis of the E-pool, 152,528 SLAFs were screened from 5,720,671 reads in each genotype with an average coverage of 37.51-fold (Table 3). From the 153,423 SLAF tags, 9,956 polymorphic SLAF were obtained. A distribution diagram of the markers on each chromosome was drawn according to the results of SLAF positioning on the genome (Fig. 3). After read-depth and quality filtration, only 1,911 high-quality and polymorphic SNPs remained for subsequent SNP-index and Δ(SNP-index) calculation. In the visualization of Δ(SNP-index) (Additional file 6), one sharp peak was observed on B05 with the Δ(SNP-index) > 0.5823, which was concentrated in the 4.08 Mb regions on B05.

**Table 3:** Summary of SLAF numbers and marker depths

Sample ID	SLAF number	Total depth	Average depth ( ×)
Jihua5	150,080	2,673,407	17.81
M130	150,190	3,355,918	22.34
P-pool	153,081	6,595,001	43.08
E-pool	152,528	5,720,671	37.51

### Genome-wide association mapping for growth-related traits in U.S. mini-core collection

In this study, the 103 peanut germplasm accessions with 12,342 (MAF > 0.05) SNPs were used to perform the association analysis. The quantile–quantile (QQ) plot showed that the GLM model (considering PCA) was suitable for reducing the number of false positive results (Additional file 7 and Additional file 8). Therefore, we conducted the GWAS for the growth-related traits with the GLM + PCA model. A total of 91 associated SNPs was detected in two locations (Additional file 9). These SNPs were located on chromosomes A01, A02, A03, A04, A05, A06, A07, A09, A10, B04, B05, B06, B07, B08, and B10.

The nineteen SNPs were identified for LBA on chromosomes A01, A04, A05, A06, A09, A10, B04, B05, and B06, and their PVE values ranged from 8.66% to 14.36%. Among these SNPs, there was one significant SNP on B06 with 14.36% PVE, and the eighteen suggestive SNPs distributed on different chromosomes. Chromosomes A04 and B06 had more associated SNPs than other chromosomes, with 8 and 3 SNPs, respectively. In addition, all three loci for LBA were located close together in B06.

A total of the 16 suggestive SNPs were detected on chromosomes A05, B05, B06, and B07 for ER. There were 10 SNPs on B07 in a genomic region from 11,291,810 to 20,276,565 with the PVE ranging from 13.79% to 14.55%. On B06, there were 2 SNPs at positions 2,362,556 and 135,069,925, respectively. The locus AX-147254196 in B06 was also detected in LBA. For IOPT, the six SNPs were identified, including five significant SNPs on A04, A09, B04, B14 and B10, and one suggestive SNP on A02 with the PVE ranging from 10.44% to 21.35%. Moreover, two SNPs were close in B04.

Thirty-eight and 12 associated SNPs were detected for MSH and LBL, respectively. The 38 significant SNPs for MSH dispersed on nine chromosomes contributed 10.88% to 17.14% of PVE. Among 12 SNPs for LBL, AX-176798127, AX-176797149, and AX-176792618 were also associated with ER, and AX-147254196 was identified related to LBA, ER, and LBL.

### Candidate genes associated with SNPs

Within the 160 kb of suggestive and significant SNPs, a total of 597 candidate genes were identified, among them 113 were for LBA, 203 for MSH, 90 for LBL, 123 for ER, and 68 for IOPT (Additional file 10), respectively. These candidate genes were distributed on A01, A02, A03, A04, A05, A06, A07, A09, A10, B05, B06, B07, B08, B09, and B10. There were more genes detected in the A subgenome than in the B subgenome. Among these genes, 66 genes were associated with plant growth (Additional file 11). Twenty-nine genes coding for the F-box protein or F-box protein interaction domain protein may be involved in the degradation of cellular proteins. Twelve genes coding for the zinc finger protein were found to have a response to light and phytohormones. Three and seven genes coding the MADS-box transcription factor were identified on the A and B subgenomes, respectively. Seven *bHLH* genes, one *WRKY* gene, and one *bZIP* gene that were involved in plant growth were also detected in associated analysis. In addition, two genes, *Aradu.BYT1F* and *Araip.WX8L5*, code for the cytochrome P450 superfamily protein; *Aradu.72XAG* and *Araip.MB9LT* code for the GATA transcription factor; and *Araip.V0CRV* and *Aradu.3X0HY* code for the FRIGIDA-like protein.

### Candidate gene validation

**Fig. 4:**
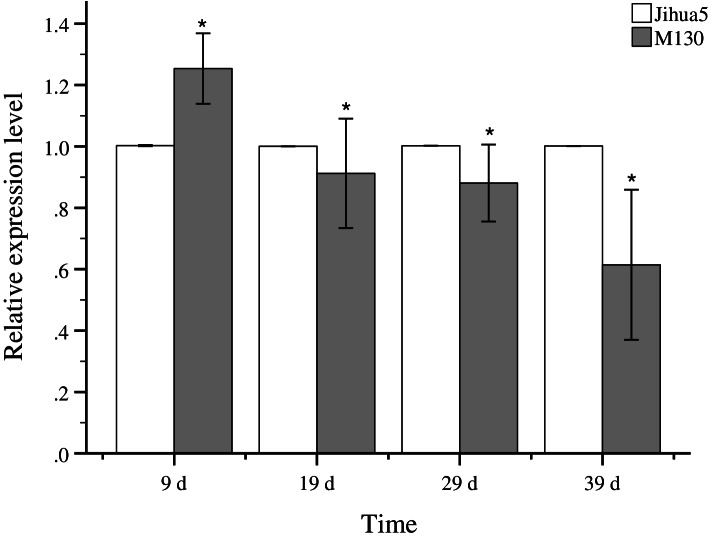
Expression levels of *Araip.E64SW* between Jihua5 and ‘M130’. Error bars represent the mean ± SD. Each data point was obtained from three biological and technical replicates. Asterisks on the top of the bars indicate statistically significant differences between Jihua5 and ‘M130’ (*0.01 < *P* < 0.05)

The identified candidate gene *Araip.E64SW* was selected to validate the gene expression level between ‘Jihua 5’ (erect) and ‘M130’ (prostrate). As shown in Fig. 4, the expression level of this gene detected by qRT-PCR showed significant difference between ‘Jihua 5’ and ‘M130’. For instance, the expression level of ‘M130’ was significantly higher than that of ‘Jihua 5’at the day 9, after that, the expression level of ‘M130’ was gradually decreased from day nine to day 39, while the expression level of Jihua maintained steadily.

## Discussion

Plant architecture is the three-dimensional organization of the aerial portion of a plant, which is strictly controlled by genetics [38]. It is mainly governed by the angle of branches, the number and length of the branches, plant height, and the structure of reproductive organs [39]. These traits determine the variety adaptability, planting patterns, mechanized harvesting, disease resistance, and potential yield. There are four market types of peanut plants: Spanish (bunch), Virginia (bunch), Virginia (runner), and Valencia (bunch). In the Spanish type, the plants grow erect with pods produced in clusters mainly around the taproot. For the bunch and runner types, the branches elongate either partially or completely along the surface of the soil. The flowering period is also considerably shorter in bunch type cultivars than in spreading type ones. Hence, the growth habit of peanut is of major agronomic importance. In this study, we combined GWAS and BSA-seq to find the loci associated with the peanut growth habit-related traits in the U.S. mini-core collection and an F_2_ population.

The core collection of germplasm resources has been developed to represent the whole germplasm collection in most crop species, such as maize [40], rice [41], and sorghum [42]. Peanut mini-core collections were established to represent the genetic diversity within the much larger germplasm collections and to provide manageable resources to conduct field studies. For peanut, three separate peanut mini-core collections comprising of 298, 112, and 184 accessions were established in China, the United States, and India, respectively [43–45]. Jiang et al. used 109 simple sequence repeat markers to genotype the 298 accessions in Chinese peanut mini-core collection and performed GWAS for 15 agronomic traits [46]. The phenotypic and molecular dissection for peanut high oleic acid can be found in the ICRISAT mini-core collection by Mukri [47]. To date, there are no reports involving GWAS of peanut growth habit-related traits using the U.S. mini-core collection, therefore the results of this study provide an important foundation for study of peanut growth habit-related traits as well as the potential to use these associated markers in the genetic improvement of peanut. The U.S. mini-core collection was utilized in this study, presenting four botanical peanut varieties (var. *fastigiata*, var. *hypogaea*, var. *vulgaris*, and var. *peruviana*) but the other two botanical varieties (var. *aequatoriana* and var. *hirsuta*) were not included [4]. Adding the addition of these two botanical varieties into the U.S. mini-core collection would enhance the genetic diversity, increase panel size, and provide a more comprehensive subset to the entire U.S. peanut germplasm collection [4; 36–37].

We first performed GWAS of growth habit-related traits on the peanut Affymetrix version 2.0 SNP array with the U.S. mini-core collection. By phenotyping the 103 accessions of the U.S. peanut mini-core collection in two environments, large phenotypic variation and significant differences among genotypes and environments were observed for the peanut growth habit-related traits. Positive correlations were observed between the LBA, LBL, ER, and IOPT, while a negative correlation was observed between LBA and MSH. These results suggested that a spreading plant type tended towards a shorter plant height. A total of 12,342 SNPs with an average of 5.19 per Mb were detected in the whole genome, and the average PIC was 0.28. It was higher than that of 0.19 [48] but lower than that of 0.53 [4] and 0.44 [36]. The panel was classified into four groups based on population structure, PCA, and phylogenetic network analysis. The results from this structure corresponded to the previous study that was constructed by using SSR markers [4]. Otyama et al. [48] separated the mini-core collection into four or five groups by using SNPs marker from a 58 K SNP array data. Moreover, the LD decay limits the mapping resolution of GWAS. Cao et al. [49] applied the 30,000 SNPs that were identified from 298 soybean accessions to evaluate the LD level and found that the mean LD (r^2^) declined to 0.2 within 360 Kb. Sun et al*.* [50] found that the LD decay was approximately 0.82 Mb in the 719 diverse accessions of upland cotton, where the r^2^ drops to the half the maximum value. Recently in peanut, Otyama et al. [48] detected the LD decay distance at 3.78 Mb, where the r^2^ dropped to half the maximum value. Based on an LD decay value of 150-160 kb (r^2^ = 0.2) in our study, the entire cultivated peanut genome (~ 2.7 G) will require 16,875–18,000 evenly spaced markers for a comprehensive GWAS evaluation. To reduce false-positive SNPs associated with these traits, two models have been developed, including the GLM-PCA and MLM-PCA-K. Although the MLM with either the PCA + K or Q + K model has been demonstrated as a successful method for identifying associations by many studies [11, 51, 52], we found the GLM-PCA was more suitable to the evaluation of population by comparing it to the MLM-PCA model (Additional file 7 and Additional file 8).

The accuracy of the GWAS results was affected by many factors, including sample size, incomplete genotyping, genetic heterogeneity, and genetic background [53]. The best way to validate the reliability of GWAS results is by using at least two methodologies. One method is validating the QTLs associated with the trait in different populations, and the other method of mutual validation is currently achieved by combining association mapping and linkage mapping in RIL or F_2_ populations, or integrating association mapping and transcriptome analysis, or BAS-seq. Han et al. performed QTL mapping and GWAS analysis associating capsaicin content in *Capsicum* using two RIL populations and one GWAS population and identified 10 co-localized QTLs [54]. Zhao et al. validated a major QTL in maize for cadmium accumulation through QTL mapping and GWA study [55]. Li et al. identified a locus for seed shattering in rice by combining BSA with a GWAS evaluation [56]. In this present study, we combined GWAS and BSA-seq associated analysis to identify candidate genes associated with LBA in peanut. The same locus on chromosome B05 in the peanut genome was mapped using GWAS and the NGS-assisted BSA approach. For BSA-seq, a 4.08 Mb physical map interval (142,610,834–146,688,220) on B05 was identified to be significantly associated with LBA. It was noteworthy that a SNP (AX-147251085) associated with LBA was detected in the same region on B05 144,353,467 in 103 peanut mini-core collection with GWAS. Based on the LD decay distance, we narrowed down and confirmed the region in 160 KB (144,193,467–144,513,467) on B05. A comprehensive analysis around the SNP (approximately 80 kb upstream and downstream) and using an annotation of the reference genome *Arachis ipaensis* identified the annotated genes in this genome region containing four candidate genes associated with the F-box family protein (*Araip.E64SW*, *Araip.YG1LK*, and *Araip.JJ6RA*) and pentatricopeptide repeat (PPR) super family protein (*Araip.YU281*) which have been shown to be important in plant growth and development [57, 58]. In this study, the expression levels of *Araip.E64SW* in prostrate plant type materials were significantly lower at 19^th^, 29^th^, and 39^th^ day, indicating the gene universally plays a negative role in regulation of the horizontal growth of branches. For ‘Jihua 5’, there was little change in the expression levels of this gene. However, it showed a downward trend in ‘M130’. Hence, the inhibition of *Araip.E64SW* may enhance the creeping growth of the first pair of lateral branches.

Peanut growth habit is a complex agronomic trait. To understand the genetic architecture of this comprehensive characteristic, the peanut growth habit traits were decomposed into five related traits, including LBA, MSH, LBL, ER, and IOPT. Among these traits, ER and LBL were strongly positively correlated with one another, with correlation coefficients of 0.79 and 0.63 within the environments of Qingyuan, Baoding, China and Dawson, GA, USA, respectively. The significant phenotypic correlation between ER and LBL could account for the four co-localization SNPs, which are AX-176798127 on chromosome A05 with 14.60% PVE, AX-176797149 with 14.60% PVE and AX-176792618 with 14.04% PVE on chromosome B05, and AX-147254196 with 19.28% PVE on chromosome B06. However, the instability of environment for the growth habit-related traits made it difficult to detect overlapping QTLs under a small number of environments [59].

A total of 66 annotated candidate genes were identified underlying the associated QTLs in the U.S. mini-core collection using the GWAS method. Among these annotated genes (Additional file 11), several genes encoded the transcription factors mediating plant growth and developmental processes, which included the *bHLH* family [60], *bZIP* family [61], *WRKY* family [62], *MADS-box* family [63], and *GATA* family [64]. In addition, we detected some genes encoding a zinc finger family protein, such as the C_2_-H_2_ zinc finger protein, which is involved in various biological processes, including hormone signaling, growth, and development [65]. The two genes, *Aradu.BYT1F* and *Araip.WX8L5,* encode the Cytochrome P450 superfamily protein, which is the largest enzymatic protein family in plants related to growth and developmental signals [66]. In addition, the genes coded by *Aradu.61ZU5* on A01*, Araip.K5RKY* on B08, and *Araip.H00Y0* and *Araip.DEM20* on B07 were associated with a FAR1-Related sequence, which plays multiple roles in light signal transduction, circadian clock, photomorphogenesis, and shoot meristems [67]. Previous studies showed that the spreading/bunch type of peanut growth habit was controlled by one locus on B05 (145,553,897 ~ 146,6459,943 bp), a putative gene associated with a FAR1-Related sequence [23]. Moreover, we constructed a high-density genetic map and co-localized 12 QTLs for growth habit-related traits on B05 (159,819,755 ~ 159,987,803 bp). However, the SNP (AX-147251085) associated with LBA was identified at position 144,353,467 on B05 in this study. Although the physical regions had no overlap, these three regions were within a megabase from each other and provide a genetic link for further map-based cloning. Furthermore, we also found some QTLs distributed on different chromosomes with high PVE for the growth habit-related traits. Overall, the candidate genes identification provides possible molecular targets but complex interactions with many biological factors such as percentage of each effector, sample size, multiple alleles, strong or weak associations, degrees of linkage disequilibrum, and the degree of correlation using a GWAS model. Therefore, the candidate genes must be validated with quantitative (q)RT-PCR. Overall, our study provides efficient strategies for detecting QTLs for growth habit-related traits in peanuts, and these findings will facilitate the development of agronomically-beneficial plant architecture to enhance peanut production.

## Conclusion

In this study, the SNP (AX-147,251,085 and AX-144,353,467) associated with LBA by GWAS was validated by the results of BSA-seq through combined analysis of GWAS and BSA-seq. Based on LD decay distance, the genome range related to LBA on B05 was shortened to 144,193,467–144,513,467. Three candidate genes related to F-box family proteins (*Araip.E64SW*, *Araip.YG1LK* and *Araip.JJ6RA*) and one candidate gene related to PPP family proteins (*Araip.YU281*) may be involved in plant growth and development. The expression analysis revealed that *Araip.E64SW* is involved in peanut growth habits. These candidate genes will provide molecular targets in marker assisted selection for peanut growth habits.

## Methods

### Plant materials and phenotyping for growth habit-related traits

A total of 103 accessions of the U.S. mini-core collection were planted in Dawson, Georgia, USA (N31°46′ and W84°26′) and Qingyuan, Baoding, China (N39°99′ and E118°70′) in 2018. The seeds of 103 accessions of the U.S. peanut mini-core collection originally came from the USDA-ARS Peanut Germplasm Collection at Griffin, GA, USA and the accessions were purified by Dr. Chen at Auburn University [68]. All these materials were granted permission. The experimental research on plants including field investigation and sample collection were performed under institutional guidelines in accordance with local legislation. These accessions were grown in a randomized complete block design with two replications. Three plants from each plot were selected to investigate the lateral branch angle (LBA), main stem height (MSH), lateral branch length (LBL), extent radius (ER), and the index of plant type (IOPT). We used the electronic protractor to measure the LBA, which is the angle between the main stem and the first lateral branch. The other traits were measured using a measuring tape, and the standards of measurement are as follows: MSH is the length from the meristematic region of the first pair lateral branches on the main stem to the internode of the parietal lobe; LBL is the length from the junction with the main stem to the parietal lobe of the longest first lateral branch; ER is the longest distance between the main stem and the first lateral branch; and IOPT is the ratio of the longest branch of the first pair lateral branches to main stem height.

An F_2_ population developed from the cross of ‘Jihua 5’ × ‘M130’ was used for bulked segregant analysis. The female parent, ‘Jihua 5’, is an erect growth habit peanut variety, and its LBA, LBL, and ER are significantly below that of male parent ‘M130’, which has a prostrate growth habit. The F_2_ population was grown in Qingyuan, Baoding, China (N39°99′ and E118°70′) for phenotyping. ‘Jihua 5’, ‘M130’ and F_2_ were originally possessed by Hebei Agricultural University.

### Genome wide association analyses for growth habit-related traits

#### DNA extraction, genotyping, and SNP screening

The genomic DNA of 103 accessions from the U.S. mini-core collection was extracted from young healthy leaves for genotyping using the CTAB method [69]. The genotyping was performed using an SNP array (Affymetrix) from GeneSeek (Lincoln, Nebraska, USA). No samples were excluded due to low quality or low call rate (< 0.95). The high-quality SNPs retained after filtering had a calling rate < 0.95 and minor allele frequency (MAF) < 0.05.

#### Population structure and association mapping analysis

The genetic structure of the U.S. mini-core collection based on polymorphic SNPs was analyzed in STRUCTURE v2.3.4. Ten independent runs were performed using the following parameters: k value of 1 to 10, a burn-in period of 10,000, and 100,000 Markov Chain Monte Carlo (MCMC) replications. The Q matrix was calculated in STRUCTURE v2.3.4. TASSEL 5.0 software was used to determine the PCA as well as the association between SNPs and phenotypic traits using a general linear model (GLM) with PCA. The LD parameter (r^2^) between pairwise SNPs (MAF > 0.05) was estimated using PopLDdecay (https://github.com/BGI-shenzhen/PopLDdecay). The threshold of suggestive and significant association between a trait and the SNPs in the GLM was p < 1.0 × 10^−3^ [i.e., − log10(p) = 3.0] [11, 70] and P < 1 × 12,342^−1^ [i.e., − log10(p) = 4.09] [15, 71]. The significance threshold was based on the Bonferroni correction for multiple tests (1/n, where n was the total number of SNPs used in the association analysis), and the GWAS results were visualized with Manhattan plots using the qqman package in R software [72].

## Bulked-segregant analysis for the growth habit-related trait

### DNA extraction, SLAF Libraries construction, and high-throughput sequencing

Genomic DNA was extracted using the modified CTAB method from fresh leaves of the ‘Jihua 5’, ‘M130’, and F_2_ populations (35 with prostrate growth habit and 35 with erect growth habit), which were used for BSA-seq [69]. Four DNA pools were constructed: the P_1_ pool from the 20 ‘Jihua 5’ plants, the P_2_ pool from the 20 ‘M130’ plants, the prostrate pool (P-pool) from the 35 extreme prostrate plants, and the erect pool (E-pool) from the 35 extremely erect plants of the F_2_ generation. DNA from these four pools was digested to completion with HaeIII and RsaI (NEB, Nanjing, China). A single-nucleotide A overhang was added to the digested fragments with Klenow Fragment (3′- 5′ exo-) (NEB, Nanjing, China) and dATP at 37℃. The duplex Tag-labeled sequencing adapters (PAGE-purified, Life Technologies, Gaithersburg, MD, USA) were ligated to the A-tailed DNA with T4 DNA ligase. Polymerase chain reaction (PCR) was performed using diluted shearing-ligation DNA samples, dNTP, Q5® High-Fidelity DNA Polymerase, and PCR primers. The PCR products were then purified using Agencourt AMPure XP beads (Beckman Coulter, High Wycombe, UK). Fragments ranging from 300 to 500 base pairs (with barcodes and adaptors) in size were excised and purified using a QIAquick gel extraction kit (Qiagen, Hilden, Germany). Gel-purified products were then diluted. Paired-end sequencing with read lengths of 125 bp was performed using an Illumina HiSeq 2500 system (Illumina, Inc., San Diego, CA, USA) according to the manufacturer’s recommendations at Beijing Biomarker Technologies Corporation (http://www.biomarker.com.cn).

### Analysis of SLAF-seq data to identify the genomic regions for growth habit-related traits

The barcodes and the terminal 5-bp positions were trimmed from each high-quality read, and clean reads from the same sample were mapped onto the *A. duranensis* and *A. ipaensis* genome sequence using SOAP software [73]. SNP and Insertions/Deletions (InDels) were detected using the software GATK [74]. To discover the genomic regions for SNPs associated with growth habit-related traits, the association analysis method of SNP-index was used [28]. The SNP-index and the Δ(SNP-index) values were calculated as follows:

SNP-index(P) = M_R_/(P_R_ + M_R_), SNP-index(E) = M_E_/(P_E_ + M_E_), Δ(SNP-index) = SNP-index(R) – SNP-index(E), where M_R_ is the depth of the R population derived from M(maternal parent), and P_R_ is the depth of the R population derived from P; M_E_ indicates the depth of the E population derived from M, and P_E_ indicates the depth of the E population derived from P.

### Candidate genes confirmation

Based on LD decay, the predicted genes around the suggestive and significant SNPs within the 160 kb and annotations of diploid ancestors of cultivated peanut, *A. duranensis* and *A. ipaensis*, were downloaded from the PeanutBase (https://peanutbase.org/home).

### Quantitative real-time PCR analysis

To validate the expression levels of candidate genes between prostrate and erect accessions, the identified candidate gene, *Araip.E64SW,* was selected to perform the quantitative real-time PCR (qRT-PCR) analysis. The ‘Jihua 5’ (erect) and ‘M130’ (prostrate) were used for this study. Fresh first pair of lateral branch were collected at 9^th^, 19^th^, 29^th^, and 39^th^ day after planting. The procedure of total RNA extraction, cDNA synthesis, qRT-RCR amplification, and candidate genes expression analysis were used as previously described [75], in which the amplification program was set as follows: 95℃ for 5 min followed by 40 cycles of 95℃ for 10 s and 60℃ for 30 s, 95℃ for 15 s, and 60℃ for 60 s. Three biological and technical repetitions were used for gene sample. The gene-specific primers were designed by Primer 5 (Additional file 12). The housekeeping gene ADH3 was used as an internal control gene for qRT-PCR normalization.

## Supplementary Information

**Additional file 1: **Analysis of variance for five traits in U.S. mini-core collectionunder two environments.

**Additional file 2: **Correlation analysis between growth habit-related traits in two environment. A. Correlation analysis for five traits in Qingyuan satation. B. Correlation analysis for five traits in Dawson. LBA, Lateral Branch Angle; MSH, Main Stem Height; LBL, Lateral Branch Length; ER, Extent Radius; IOPT, the Index of Plant type. ** representing significance at *P* < 0.01 level (two-tailed). *representing significance at *P* < 0.05 level(two-tailed). 

**Additional file 3: **One hundred and three genotypes mainly coming from the peanut mini-core collection used for GWAS analysis related with growth habit-related traits. 

**Additional file 4:** The distribution frequency of each subgroup within each botanical variety (A), and each botanical variety within each subpopulation (B).

**Additional file 5:** The value of genome-wide average LD decay. The x-axis indicates the inter-marker genetic distance and the y-axis indicates the r2 value.

**Additional file 6: **The result of marker SNP index associated with LBA. The x-axis indicates the position of the chromosome and the y-axis indicates the value of the ΔSNP index. Black lines indicated that the result of all Della SNP index after fitting. The dotted line showed that the threshold value of the ΔSNP index. 

**Additional file 7:** Manhattan plots along with QQ plots showing the GWAS for growth habit-related traits by GLM in Qingyuan station. The dashed horizontal line represents the significance threshold (*P* < 1×12342−1) and suggestive line (*P*< 1.0 × 10−3).

**Additional file 8:** Manhattan plots along with QQ plots showing the GWAS for growth habit-related traits by GLM in Dawson station. The dashed horizontal line represents the significance threshold (*P* < 1×12342−1) and suggestive line (*P*< 1.0 × 10−3).

**Additional file 9:** The summary of SNPs associated with growthhabit-related traits.

**Additional file 10:** List of 597 candidate genes with growth habit-related traits in peanuts.

**Additional file 11: **List of  transcription factors mediating plant growth and developmental processes.

**Additional file 12:** Primers used for qRT-PCR.

## Data Availability

The data that support the findings of this study are openly available on the. SRA database under Bioproject accession PRJNA746761 (https://www.ncbi.nlm.nih.gov/bioproject/PRJNA746761).

## Abbreviations

QTL: Quantitative trait loci
GWAS: Genome-wide association analysis
BSA-seq: Bulked segregant analysis sequencing
SNP: Single nucleotide polymorphism
LBA: Lateral branch angle
MSH: Main stem height
LBL: Lateral branch height
ER: Extent radius
IOPT: Index of plant type
SLAF-seq: Specific length amplified fragment sequencing
LD: Linkage disequilibrium
RIL: Recombinant inbred lines
QQ: Quantile-quantile
MAF: Minor allele frequency
InDels: Insertions/Deletions
