# Supplementary material for: GWAS and bulked segregant analysis reveal the Loci controlling growth habit-related traits in cultivated Peanut (Arachis hypogaea L.)
Source: BMC Genomics. 2022 May 27;23:403. doi: 10.1186/s12864-022-08640-3 (PMC9145184; doi:10.1186/s12864-022-08640-3)
Supplement: Supplementary file 1 — Additional file 1: Analysis of variance for five traits in U.S. mini-core collectionunder two environments. [file 12864_2022_8640_MOESM1_ESM.pdf]

Additional file 1. Analysis of variance for five traits in U.S. mini-core collection under two environments

Traits	Variables	df	Sum of Squares	Mean Square	*F* value
LBA	Genotype	102	39752.912	389.734	34.705**
	Environments	1	140.184	140.184	12483**
	Genotype × environments	100	11899.837	118.998	10.597**
	Error	204	2290.878	11.23	
MSH	Genotype	102	19067.193	186.933	18.342**
	Environments	1	40539.52	40539.52	3977.674**
	Genotype × environments	100	7978.028	79.78	7.828**
	Error	204	2079.12	10.192	
LBL	Genotype	102	22080.401	216.475	18.723**
	Environments	1	51245.839	51245.839	4432.349**
	Genotype × environments	100	10260.709	102.607	8.875**
	Error	204	2358.603	11.562	
ER	Genotype	102	14199.319	139.209	21.485**
	Environments	1	4115.484	4115.484	635.175**
	Genotype × environments	100	5463.745	54.637	8.433**
	Error	204	1321.775	6.479	
IOPT	Genotype	102	69.237	0.679	17.896**
	Environments	1	9.728	9.728	256.462**
	Genotype × environments	100	29.889	0.299	7.88**
	Error	204	7.738	0.038	

*, ** Significant differences at $P < 0.05$ and $P < 0.01$, respectively.
