# Supplementary material for: GWAS and bulked segregant analysis reveal the Loci controlling growth habit-related traits in cultivated Peanut (Arachis hypogaea L.)
Source: BMC Genomics. 2022 May 27;23:403. doi: 10.1186/s12864-022-08640-3 (PMC9145184; doi:10.1186/s12864-022-08640-3)
Supplement: Supplementary file 2 — Additional file 2: Correlation analysis between growth habit-related traits in two environment. A. Correlation analysis for five traits in Qingyuan satation. B. Correlation analysis for five traits in Dawson. LBA, Lateral Branch Angle; MSH, Main Stem Height; LBL, Lateral Branch Length; ER, Extent Radius; IOPT, the Index of Plant type. ** representing significance at P < 0.01 level (two-tailed). *representing significance at P < 0.05 level(two-tailed). [file 12864_2022_8640_MOESM2_ESM.pdf]

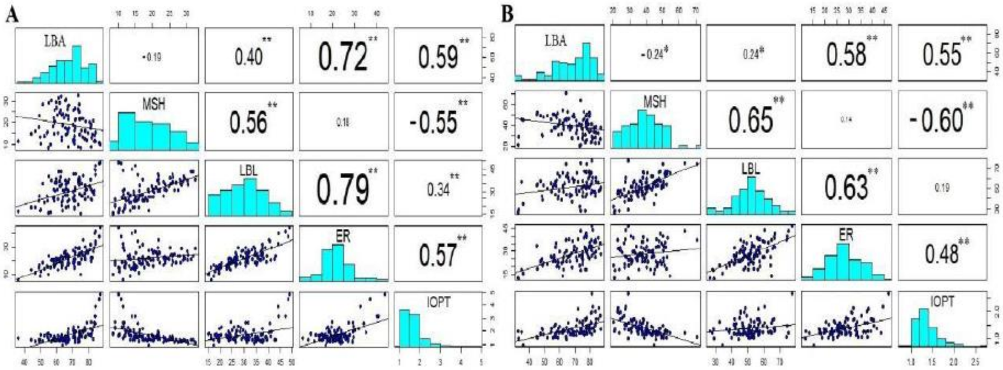

Additional file 2. Correlation analysis between growth habit-related traits in two environment. A. Correlation analysis for five traits in Qingyuan satation. B. Correlation analysis for five traits in Dawson. LBA, Lateral Branch Angle; MSH, Main Stem Height; LBL, Lateral Branch Length; ER, Extent Radius; IOPT, the Index of Plant type. ** representing significance at $P < 0.01$ level (two-tailed). *representing significance at $P < 0.05$ level(two-tailed).
