# Supplementary material for: GWAS and bulked segregant analysis reveal the Loci controlling growth habit-related traits in cultivated Peanut (Arachis hypogaea L.)
Source: BMC Genomics. 2022 May 27;23:403. doi: 10.1186/s12864-022-08640-3 (PMC9145184; doi:10.1186/s12864-022-08640-3)
Supplement: Supplementary file 3 — Additional file 3: One hundred and three genotypes mainly coming from the peanut mini-core collection used for GWAS analysis related with growth habit-related traits. [file 12864_2022_8640_MOESM3_ESM.pdf]

Additional file 3. One hundred and three genotypes mainly coming from the peanut mini-core collection used for GWAS analysis related with growth habit-related traits

Code	PI no.	Botanical variety	Origin
A-001	PI290560	*vulgaris*	India
A-002	PI290620	*fastigiata*	Argentina
A-003	PI290566	*fastigiata*	India
A-004	PI290594	*hypogaea*	India
A-005	PI290536	*hypogaea*	India
A-006	PI343398	*fastigiata*	Israel
A-007	PI343384	*hypogaea*	Israel
A-008	PI371521	*hypogaea*	Israel
A-009	PI200441	*fastigiata*	Japan
A-010	PI196635	*hypogaea*	Madagascar
A-011	PI259851	*hypogaea*	Malawi
A-012	PI355271	*hypogaea*	Mexico
A-013	PI372271	*hypogaea*	Unknown
A-014	PI399581	*hypogaea*	Nigeria
A-015	PI337406	*fastigiata*	Paraguay
A-016	PI159786	*hypogaea*	Senegal
A-017	PI268696	*hypogaea*	South Africa
A-018	PI298854	*hypogaea*	South Africa
A-019	PI268868	*hypogaea*	Sudan
A-020	PI313129	*fastigiata*	Taiwan
A-021	PI155107	*vulgaris*	Uruguay
A-022	PI162655	*hypogaea*	Uruguay
A-023	PI152146	*fastigiata*	Uruguay
A-024	PI262038	*fastigiata*	Brazil
A-025	PI337293	*hypogaea*	Brazil
A-026	PI270907	*hypogaea*	Zambia
A-027	PI270905	*hypogaea*	Zambia
A-028	PI268996	*hypogaea*	Zambia
A-029	PI270998	*vulgaris*	Zambia
A-030	PI268806	*hypogaea*	Zambia
A-031	PI268755	*hypogaea*	Zambia
A-032	PI270786	*hypogaea*	Zambia
A-033	PI356004	*fastigiata*	Argentina
A-034	PI493547	*fastigiata*	Argentina
A-035	PI259658	*hypogaea*	Cuba
A-037			
