# Supplementary material for: GWAS and bulked segregant analysis reveal the Loci controlling growth habit-related traits in cultivated Peanut (Arachis hypogaea L.)
Source: BMC Genomics. 2022 May 27;23:403. doi: 10.1186/s12864-022-08640-3 (PMC9145184; doi:10.1186/s12864-022-08640-3)
Supplement: Supplementary file 4 — Additional file 4: The distribution frequency of each subgroup within each botanical variety (A), and each botanical variety within each subpopulation (B). [file 12864_2022_8640_MOESM4_ESM.pdf]

Additional file 4. The distribution frequency of each subgroup within each botanical variety (A), and each botanical variety within each subpopulation (B).
