# Supplementary material for: GWAS and bulked segregant analysis reveal the Loci controlling growth habit-related traits in cultivated Peanut (Arachis hypogaea L.)
Source: BMC Genomics. 2022 May 27;23:403. doi: 10.1186/s12864-022-08640-3 (PMC9145184; doi:10.1186/s12864-022-08640-3)
Supplement: Supplementary file 5 — Additional file 5: The value of genome-wide average LD decay. The x-axis indicates the inter-marker genetic distance and the y-axis indicates the r2 value. [file 12864_2022_8640_MOESM5_ESM.pdf]

Additional file 5. The value of genome-wide average LD decay. The x-axis indicates the inter-marker genetic distance and the y-axis indicates the r^2 value.
