# Supplementary material for: GWAS and bulked segregant analysis reveal the Loci controlling growth habit-related traits in cultivated Peanut (Arachis hypogaea L.)
Source: BMC Genomics. 2022 May 27;23:403. doi: 10.1186/s12864-022-08640-3 (PMC9145184; doi:10.1186/s12864-022-08640-3)
Supplement: Supplementary file 6 — Additional file 6: The result of marker SNP index associated with LBA. The x-axis indicates the position of the chromosome and the y-axis indicates the value of the ΔSNP index. Black lines indicated that the result of all Della SNP index after fitting. The dotted line showed that the threshold value of the ΔSNP index. [file 12864_2022_8640_MOESM6_ESM.pdf]

Additional file 6. The result of marker SNP index associated with LBA. The x-axis indicates the position of the chromosome and the y-axis indicates the value of the ΔSNP index. Black lines indicated that the result of all Della SNP index after fitting. The dotted line showed that the threshold value of the ΔSNP index.
