# Supplementary material for: GWAS and bulked segregant analysis reveal the Loci controlling growth habit-related traits in cultivated Peanut (Arachis hypogaea L.)
Source: BMC Genomics. 2022 May 27;23:403. doi: 10.1186/s12864-022-08640-3 (PMC9145184; doi:10.1186/s12864-022-08640-3)
Supplement: Supplementary file 8 — Additional file 8: Manhattan plots along with QQ plots showing the GWAS for growth habit-related traits by GLM in Dawson station. The dashed horizontal line represents the significance threshold (P < 1×12342−1) and suggestive line (P< 1.0 × 10^−3^). [file 12864_2022_8640_MOESM8_ESM.pdf]

Additional file 8. Manhattan plots along with QQ plots showing the GWAS for growth habit-related traits by GLM in Dawson station. The dashed horizontal line represents the significance threshold ($P < 1 \times 12342^{-1}$) and suggestive line ($P < 1.0 \times 10^{-3}$).
