# Supplementary material for: GWAS and bulked segregant analysis reveal the Loci controlling growth habit-related traits in cultivated Peanut (Arachis hypogaea L.)
Source: BMC Genomics. 2022 May 27;23:403. doi: 10.1186/s12864-022-08640-3 (PMC9145184; doi:10.1186/s12864-022-08640-3)
Supplement: Supplementary file 9 — Additional file 9: The summary of SNPs associated with growthhabit-related traits. [file 12864_2022_8640_MOESM9_ESM.docx]

**Additional file 9** The summary of SNPs associated with growth habit-related traits

| Trait | Marker | Chromosome | Position | allele | MAF | -log10(p) | R^2^(100%) | Environment |
| --- | --- | --- | --- | --- | --- | --- | --- | --- |
| Angle | AX-176792214 | 1 | 5676938 | A/C | 0.16 | 3.10 | 8.70% | Qingyuan |
|  | AX-147220276 | 4 | 88049958 | T/C | 0.32 | 3.29 | 11.54% | Qingyuan |
|  | AX-176812753 | 4 | 11518168 | T/C | 0.35 | 3.12 | 8.75% | Qingyuan |
|  | AX-176805320 | 4 | 11519148 | A/C | 0.30 | 3.12 | 8.75% | Qingyuan |
|  | AX-176795188 | 4 | 101622076 | A/G | 0.35 | 3.12 | 8.75% | Qingyuan |
|  | AX-176805974 | 4 | 101656655 | A/G | 0.30 | 3.12 | 8.75% | Qingyuan |
|  | AX-176800474 | 4 | 101794652 | A/G | 0.35 | 3.12 | 8.75% | Qingyuan |
|  | AX-176801418 | 4 | 58618730 | T/C | 0.35 | 3.10 | 8.78% | Qingyuan |
|  | AX-176795352 | 4 | 11517909 | T/C | 0.35 | 3.04 | 8.66% | Qingyuan |
|  | AX-147221637 | 5 | 7049419 | A/G | 0.27 | 3.26 | 14.00% | Dawson |
|  | AX-147226655 | 6 | 110138057 | A/C | 0.42 | 4.07 | 14.01% | Qingyuan |
|  | AX-147232753 | 9 | 10663409 | T/C | 0.40 | 3.21 | 11.55% | Qingyuan |
|  | AX-147235889 | 10 | 44591641 | T/C | 0.49 | 3.52 | 12.28% | Qingyuan |
|  | AX-176793547 | 14 | 64739604 | T/G | 0.34 | 3.56 | 10.89% | Qingyuan |
|  | AX-176804663 | 14 | 20502112 | T/C | 0.34 | 3.43 | 9.99% | Qingyuan |
|  | AX-147251085 | 15 | 144353467 | A/G | 0.15 | 3.42 | 9.71% | Qingyuan |
|  | AX-147254206 | 16 | 135227462 | A/G | 0.49 | 4.18 | 14.36% | Qingyuan |
|  | AX-147254196 | 16 | 135069925 | T/G | 0.49 | 3.32 | 11.76% | Qingyuan |
|  | AX-147254204 | 16 | 135176210 | A/G | 0.16 | 3.30 | 11.56% | Qingyuan |
| ER | AX-176798127 | 5 | 32468191 | T/C | 0.21 | 3.68 | 14.60% | Qingyuan |
|  | AX-147223814 | 5 | 106305542 | T/G | 0.32 | 3.00 | 13.61% | Dawson |
|  | AX-176797149 | 15 | 29316793 | T/C | 0.21 | 3.68 | 14.60% | Qingyuan |
|  | AX-176792618 | 15 | 28864664 | T/C | 0.21 | 3.50 | 14.04% | Qingyuan |
|  | AX-147254196 | 16 | 135069925 | T/G | 0.49 | 4.96 | 19.28% | Qingyuan |
|  | AX-176809834 | 16 | 2362556 | A/G | 0.15 | 3.14 | 14.20% | Dawson |
|  | AX-176806386 | 17 | 18553475 | A/G | 0.39 | 3.20 | 14.55% | Dawson |
|  | AX-176817808 | 17 | 11291810 | A/C | 0.38 | 3.05 | 13.79% | Dawson |
|  | AX-176810970 | 17 | 11421301 | A/G | 0.38 | 3.05 | 13.79% | Dawson |
|  | AX-147255025 | 17 | 11796265 | A/G | 0.38 | 3.05 | 13.79% | Dawson |
|  | AX-177638806 | 17 | 12444126 | A/G | 0.38 | 3.05 | 13.79% | Dawson |
|  | AX-177637880 | 17 | 12758016 | A/G | 0.38 | 3.05 | 13.79% | Dawson |
|  | AX-176818695 | 17 | 17400993 | A/G | 0.39 | 3.05 | 13.79% | Dawson |
|  | AX-177644547 | 17 | 17400993 | A/G | 0.39 | 3.05 | 13.79% | Dawson |
|  | AX-177638883 | 17 | 18950781 | A/G | 0.39 | 3.05 | 13.79% | Dawson |
|  | AX-176798120 | 17 | 20276565 | A/G | 0.39 | 3.05 | 13.79% | Dawson |
| IOPT | AX-147221270 | 4 | 120697576 | T/G | 0.47 | 5.83 | 18.59% | Qingyuan |
|  | AX-176803063 | 9 | 291318 | A/G | 0.07 | 6.87 | 21.35% | Qingyuan |
|  | AX-147248798 | 14 | 130877416 | A/C | 0.47 | 5.91 | 18.75% | Qingyuan |
|  | AX-147248787 | 14 | 130856189 | T/C | 0.48 | 4.61 | 15.07% | Qingyuan |
|  | AX-176823682 | 20 | 123110042 | A/G | 0.17 | 4.25 | 16.08% | Dawson |
|  | AX-147213787 | 2 | 52026360 | A/G | 0.48 | 3.31 | 10.44% | Dawson |
| LBL | AX-176798127 | 5 | 32468191 | T/C | 0.21 | 4.28 | 16.71% | Qingyuan |
|  | AX-147223780 | 5 | 105764065 | T/C | 0.14 | 3.48 | 11.24% | Qingyuan |
|  | AX-176796238 | 5 | 104023520 | A/G | 0.18 | 3.48 | 10.72% | Qingyuan |
|  | AX-176814912 | 5 | 62228147 | A/G | 0.30 | 3.13 | 12.54% | Qingyuan |
|  | AX-176822343 | 5 | 59674162 | T/C | 0.31 | 3.08 | 12.48% | Qingyuan |
|  | AX-176797149 | 15 | 29316793 | T/C | 0.21 | 4.28 | 16.71% | Qingyuan |
|  | AX-176792618 | 15 | 28864664 | T/C | 0.21 | 4.27 | 16.82% | Qingyuan |
|  | AX-176810022 | 15 | 145958219 | T/C | 0.15 | 3.74 | 12.16% | Qingyuan |
|  | AX-147250474 | 15 | 129381149 | A/G | 0.20 | 3.20 | 12.94% | Dawson |
|  | AX-176819482 | 15 | 126045337 | A/C | 0.19 | 3.11 | 12.59% | Dawson |
|  | AX-147254196 | 16 | 135069925 | T/G | 0.49 | 4.24 | 16.70% | Qingyuan |
|  | AX-176821018 | 19 | 128388128 | A/C | 0.11 | 3.00 | 9.71% | Dawson |
| MSH | AX-176805993 | 3 | 124706141 | A/C | 0.34 | 4.78 | 13.26% | Qingyuan |
|  | AX-176812621 | 4 | 27220270 | A/G | 0.10 | 5.71 | 17.14% | Dawson |
|  | AX-176803436 | 4 | 43672731 | A/C | 0.10 | 5.71 | 17.14% | Dawson |
|  | AX-176811509 | 4 | 73585852 | A/G | 0.10 | 5.03 | 15.36% | Dawson |
|  | AX-147220444 | 4 | 101534011 | T/C | 0.10 | 4.78 | 14.68% | Dawson |
|  | AX-177638120 | 7 | 66573812 | T/G | 0.29 | 5.27 | 14.62% | Qingyuan |
|  | AX-177638387 | 7 | 65115370 | A/G | 0.29 | 5.07 | 13.97% | Qingyuan |
|  | AX-147228341 | 7 | 45942702 | A/G | 0.19 | 4.49 | 14.62% | Qingyuan |
|  | AX-176802722 | 7 | 50745863 | T/G | 0.19 | 4.49 | 13.97% | Qingyuan |
|  | AX-176804668 | 7 | 54958888 | T/G | 0.19 | 4.49 | 12.55% | Qingyuan |
|  | AX-177644532 | 10 | 2709148 | A/G | 0.35 | 4.65 | 11.01% | Qingyuan |
|  | AX-176820262 | 17 | 10347054 | T/C | 0.38 | 4.95 | 13.67% | Qingyuan |
|  | AX-147256096 | 17 | 105991515 | A/G | 0.38 | 4.90 | 13.57% | Qingyuan |
|  | AX-177637395 | 17 | 9176911 | T/C | 0.48 | 4.58 | 12.79% | Qingyuan |
|  | AX-176801688 | 17 | 121093831 | T/C | 0.48 | 4.32 | 12.29% | Qingyuan |
|  | AX-176799807 | 18 | 103258895 | A/G | 0.19 | 4.54 | 12.89% | Qingyuan |
|  | AX-176817932 | 18 | 96048496 | T/C | 0.19 | 4.49 | 12.55% | Qingyuan |
|  | AX-176798062 | 18 | 98749831 | T/C | 0.19 | 4.49 | 12.55% | Qingyuan |
|  | AX-176807772 | 18 | 101547178 | A/C | 0.19 | 4.49 | 12.55% | Qingyuan |
|  | AX-176817947 | 18 | 101858265 | A/G | 0.19 | 4.49 | 12.55% | Qingyuan |
|  | AX-177637253 | 18 | 103258895 | A/G | 0.19 | 4.49 | 12.55% | Qingyuan |
|  | AX-176794504 | 18 | 129311474 | T/C | 0.19 | 4.49 | 12.55% | Qingyuan |
|  | AX-176820517 | 19 | 114568297 | T/C | 0.19 | 4.49 | 12.55% | Qingyuan |
|  | AX-176804144 | 20 | 115730891 | T/C | 0.31 | 4.83 | 13.39% | Qingyuan |
|  | AX-177638677 | 20 | 120214136 | T/C | 0.31 | 4.83 | 13.39% | Qingyuan |
|  | AX-177637104 | 20 | 121402159 | T/C | 0.31 | 4.83 | 13.39% | Qingyuan |
|  | AX-176804925 | 20 | 112467768 | T/C | 0.35 | 4.60 | 10.88% | Qingyuan |
|  | AX-176822799 | 20 | 116425340 | T/C | 0.35 | 4.60 | 10.88% | Qingyuan |
|  | AX-176801688 | 17 | 121093831 | T/C | 0.48 | 4.32 | 12.29% | Qingyuan |
|  | AX-176822282 | 14 | 30221792 | A/G | 0.24 | 4.28 | 12.35% | Qingyuan |
|  | AX-176817941 | 18 | 103194207 | T/C | 0.19 | 4.16 | 11.83% | Qingyuan |
|  | AX-176807024 | 3 | 115530645 | T/C | 0.44 | 4.11 | 11.59% | Qingyuan |
|  | AX-176803234 | 7 | 43817432 | T/C | 0.44 | 4.11 | 11.59% | Qingyuan |
|  | AX-177637849 | 7 | 51512828 | T/G | 0.44 | 4.11 | 11.59% | Qingyuan |
|  | AX-176794936 | 18 | 105532652 | T/C | 0.44 | 4.11 | 11.59% | Qingyuan |
|  | AX-176802597 | 18 | 106146502 | A/G | 0.44 | 4.11 | 11.59% | Qingyuan |
|  | AX-147258684 | 18 | 106275540 | A/C | 0.44 | 4.11 | 11.59% | Qingyuan |
|  | AX-176805714 | 19 | 106118827 | T/G | 0.44 | 4.11 | 11.59% | Qingyuan |
